# Working toward Improved Chemical Covers for Water Conservation: A Laboratory-scale Assessment of Antievaporative Lipid Films

**DOI:** 10.1021/acs.langmuir.6c01906

**Published:** 2026-08-04

**Authors:** Julia Elisa Sevón, Riku Paananen, Jukka Moilanen, Tuomo Viitaja, Filip S. Ekholm

**Affiliations:** † Department of Chemistry, 3835 University of Helsinki, P.O. Box 55, FI-00014 Helsinki, Finland; ‡ Ophthalmology, University of Helsinki and Helsinki University Hospital, Haartmaninkatu 8, FI-00290 Helsinki, Finland

## Abstract

The devastating effects of drought are expected to increase as a result of the global climate change. This poses growing challenges related to water conservation and efficient consumption. Improved chemical covers designed to mitigate water evaporation from reservoirs and open water bodies represent a potential countermeasure in water conservation. In this work, we set out to perform a laboratory-scale assessment of the possibility to develop improved chemical covers based on antievaporative lipid films featuring *O*-acyl-ω-hydroxy-fatty acids, fatty alcohols, wax esters or their combinations. We utilized a Langmuir trough experimental setup in order to assess film structure, spreadability and evaporation resistance. Moreover, we were able to tailor compositions which show superior antievaporative properties compared to current state-of-the-art solutions. Altogether, the work generates a solid foundation for the future design of improved chemical covers for water evaporation mitigation.

## Graphical abstract

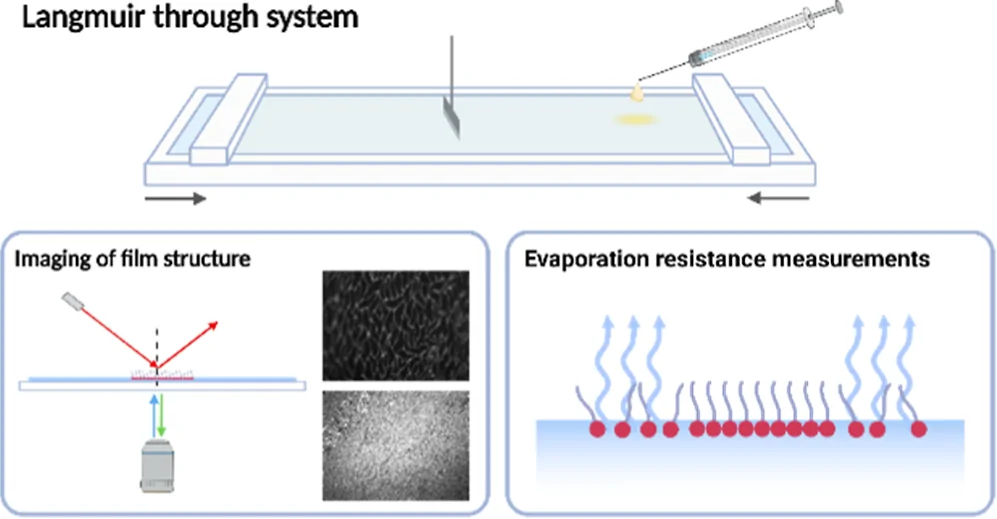

## Introduction

Drought has extensive and devastating impacts not only on ecosystems and biodiversity but also on the world economy and the functioning of society.^1^ It has traditionally affected life in arid and semiarid areas, but as the climate change progresses, the destructive effects are threatening to spread drastically. According to a United Nations Environment Programme report (UNEP, 2015), nearly half of the world’s population could suffer from water stress by 2030.^2^ As a consequence, solving challenges related to water conservation and planning efficient water consumption in the future are becoming more relevant than ever.

One of the most significant sources of water loss is evaporation from open water storages and reservoirs, such as lakes, ponds and dams. It has been estimated that approximately 25% of potable water in reservoirs is lost due to this phenomenon.^3^ The rate of water evaporation can however be reduced if the surface area of water that is in contact with air and sun radiation is minimized.^4,5^ Several distinct solutions for combating water evaporation have been tried including strategies based on physical, biological and chemical covers.

Physical covers, such as shade cloths and different types of continuous or modular floating covers, have shown to be effective in water evaporation mitigation.^5–11^ However, these methods also come with disadvantages. Physical covers are often laborious and expensive to install, requiring a substantial upfront investment.^5–7^ Another concern is the potential environmental impact of these covers, as physical covers can hinder both sunlight penetration and gas exchange, leading to potential adverse effects on water quality and local aquatic ecosystems.^7,12,13^ Biological covers on the other hand, such as floating aquatic plants or covers made from organic residue, are usually less effective than physical covers and prone to degradation.^5^ Moreover, the ecological effects of biological covers are not fully understood^14^ and as for the aquatic plants, the transpiration of the plants and their effects on the ecosystem needs to be considered. Consequently, these drawbacks limit the potential of physical and biological covers, especially in natural waters, large water bodies and multipurpose water systems.

Given these restraints, chemical covers continue to be considered as a solid alternative. Chemical covers are thin surfactant films which are capable of forming a physical diffusion barrier on top of water, thereby reducing the loss of water molecules from the surface.^15,16^ These films can be surprisingly thin, even down to a single molecular layer. In particular, long-chain fatty alcohols, specifically hexadecanol and octadecanol, have been extensively researched for evaporation mitigation due to their favorable spreading rates^16–18^ and substantial reductions in water evaporation,^19–21^ prompting subsequential studies in outdoor evaporation pans and water reservoirs.^5,17^ For instance, Tang et al.^22^ achieved a 60% reduction using octadecanol in 1.2 m diameter evaporation pans, while Panjabi et al.^23^ reported a 19.26% reduction over a six month trial conducted at the Aji Reservoir in India using a powder mixture of both alcohols. Such promising results have additionally led to the development of commercial products, such as WaterSavr, which features both hexadecanol and octadecanol and achieves a reduction in water evaporation of 30–35% in open water systems.^24^

While chemical covers can significantly reduce water evaporation, there are several challenges associated with their use. The ecological impact of chemical covers needs to be carefully considered as well as the compounds’ degradation routes driven by bacteria^25,26^ or solar radiation.^17^ Furthermore, physical challenges such as vaporization^27^ and displacement by wind flow^28,29^ can drastically reduce the efficiency of chemical covers in field environments. Consequently, the compounds often require reapplication every few days to maintain their evaporation mitigation capacity.^9,17^ This poses a significant challenge as maintenance expenses constitute a substantial portion of the total cost of the method. Additionally, various impurities in natural waters further reduce the evaporation reduction and may hinder the spreading of the compounds. As a results of these limitations, it is important that the molecular solutions utilized are able to spread and respread on the water surface to help repair disturbances in the film structure.^17^

Despite the challenges noted upon, chemical covers offer three key advantages: the strategies are economically sensible,^30^. the molecular solutions are tailorable and their adverse environmental impact may be lower than that of alternative cover options.^31,32^ Therefore, chemical covers represent a promising option for large water bodies and multipurpose water systems. Nonetheless, continued research is essential to overcome current limitations and develop solutions that offer both a more efficient evaporation reduction and a longer lifespan on the water surface.

In this work, we focus on the first stage of the development of improved molecular solutions for chemical covers, i.e. on a laboratory scale assessment (utilizing Langmuir troughs) of lipid films evaluating spreadability, film properties, and evaporation resistance. Our choice of molecular solutions to pursue is heavily inspired by the antievaporative human tear film lipid classes responsible for retardation of water evaporation in the human tear film.^33,34^ Through years of dedicated effort focused on investigation of tear film lipids through a modern chemistry-biophysics approach, we have identified two lipid classes as suitable starting points for this study: *O*-acyl-ω-hydroxy-fatty acids (OAHFAs) and wax esters (WE), including their combinations. In addition, via detailed mapping of structure–activity profiles in these classes at physiological conditions, we previously generated overarching insights on the optimal balance between structural parameters (such as chain lengths, degree of saturation and functional groups) and performance (such as spreadability and evaporation resistance) at temperatures of 35 °C.^33–35^ Therefore, a sound foundation was available for the current work, although the effects of temperature on film performance had not been previously assessed and the temperatures relevant for water conservation purposes differ from those at the ocular surface. Consequently, we expected that the development of solutions for water conservation applications would require careful revisiting and revisions to the structural parameters of the molecular solutions to be explored. Additionally, we considered it worthwhile to simultaneously evaluate the potential of optimizing existing solutions based on long-chain fatty alcohols. At the outset of the investigations, we considered these molecular solutions to be viable and promising options because: (1) these molecular classes are naturally occurring and should thus be relatively well tolerated by the environment, (2) films formed by OAHFA and OAHFA:WE-compositions have been proven to display moderate-to-exceptional antievaporative properties,^34,36^ and, (3) the synthetic routes to these species are generally short thus improving the tailorability and cost-efficiency of the approach.

## Materials and Methods

### Materials

It is well-known that the biophysical properties of OAHFAs are to a high degree temperature dependent, and therefore, three different OAHFAs with varying chain lengths were selected for the studies (Figure 1). These compounds were studied on their own and as 1:1 molar compositions together with behenyl oleate (C_22:0_/C_18:1_-WE, Figure 1), which was chosen as a representative WE based on our previous work. The OAHFAs studied herein were: C_12:0_/C_18:1_-OAHFA, C_15:0_/C_18:1_-OAHFA and C_20:0_/C_18:1_-OAHFA. These compounds are structural analogues of naturally occurring OAHFAs,^33,37–39^ but retain their functional aspects to an acceptable degree. The OAHFAs were synthesized in-house as previously described, and synthetic protocols and analytical data can be found in our previous publications.^33^ C_22:0_/C_18:1_-WE was purchased from Larodan Ltd. (Solna, Sweden) and octadecanol (utilized as a model of the long-chain fatty alcohols, Figure 1) was acquired from Sigma-Aldrich (St. Louis, MO, USA). The palmitic acid-NBD (16-NBD) used in imaging was purchased from Avanti Polar Lipids Inc. (Alabaster, AL, USA).

**Figure 1:**
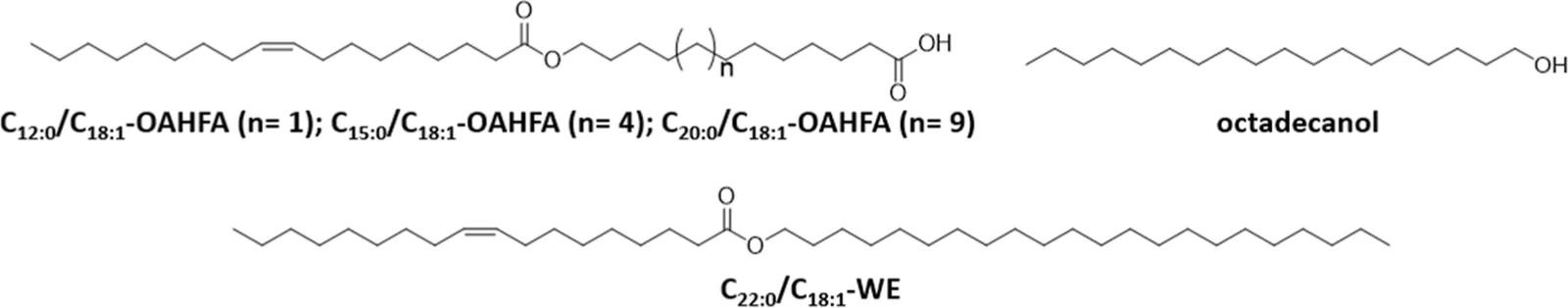
Structures of the studied compounds. Three different OAHFA species were chosen: C_12:0_/C_18:1_-OAHFA, C_15:0_/C_18:1_-OAHFA and C_20:0_/C_18:1_-OAHFA, together with C_22:0_/C_18:1_-WE, which was chosen as a representative WE, and octadecanol, which was chosen as representative long-chain fatty alcohol because it is a component in current chemical covers.

### Surface Pressure Isotherms/Imaging

The studies were conducted using a Langmuir trough system. First, we measured the surface pressure isotherms for each compound and representative compositions and these studies were accompanied by imaging of the film structure. For the pure compounds, imaging was done using fluorescence microscopy (FM), but for the compositions, Brewster angle microscopy (BAM) was also employed in order to gain additional insight on film structures. All Langmuir trough experiments were done at room temperature and Milli-Q -water was used as a subphase. The surface pressure/area isotherms were measured using a KSV large Langmuir trough (Biolin Scientific, Espoo, Finland; dimensions 580 × 145 mm), and the surface pressure was recorded using a Wilhelmy plate. To ensure a low-ozone environment and prevent oxidation of the lipids under the measurements,^40,41^ the experimental setup was enclosed in an acrylic box (volume 250 L) and dry air was continuously led into the container at a flow rate of 76 L/min via an ozone solutions ODS-3P ozone destruct unit (Hull, Iowa). The lipids were added to the subphase surface as a chloroform solution (in a typical experiment 150 nmol of lipids was added onto the subphase surface), using a 50 μL Hamilton syringe (Hamilton Central Europe S.R.L., Ghiroda, Romania) or a 50 μL eVol XR digital analytical syringe (SGE Analytical Science, Ringwood, VIC, Australia). The chloroform was allowed to evaporate for 5 min, and after that the film was compressed with a barrier speed of 10 mm/min. During the compression, changes in the surface pressure were recorded, and the isotherms for each compound/composition were measured a minimum of three times. The BAM-imaging of the lipid films was done simultaneously with the surface pressure isotherm measurements using a KSV NIMA microBAM camera (Biolin Scientific).

In order to study the lipid films with fluorescence microscopy (FM), 0.5 mol % fluorescent palmitic acid - NBD (16-NBD, Avanti Polar Lipids Inc., Alabaster, AL, USA) was added to the lipid species/compositions as such. The FM experiments were done using a Kibron Micro Trough X -Langmuir trough (Kibron, Helsinki, Finland; dimensions 59 × 208 mm), and the surface pressure was measured using a surface pressure sensor (KBN 315; Kibron) with a Kibron DyneProbe. In a typical experiment, 20 nmol of lipids were added to the subphase surface as described above, and after allowing the chloroform to evaporate, the film was compressed with a barrier speed of 10 mm/min. During the compression, the surface pressure was recorded, and the film structure was imaged with an inverted ECLIPSE TE 2000 E microscope (Nikon, Minato, Japan) with a TE 2000 T-FL Epi-Fl -attachment (Nikon) and a Fluor ELWD 20*x*/0.45 objective (Nikon). The black and white images were taken with a Digital Sight DS-5Mc camera (Nikon).

### Evaporation Resistance

The evaporation resistances were measured using the KSV large Langmuir trough setup described above. The lipids were added to the subphase surface as described previously, and after that, the film was compressed with a barrier speed of 20 mm/min. The compression was paused at chosen areas (ranging between 25 and 3.5 Å^2^/molecule) and the evaporation resistance was determined at these areas using the Langmuir–Schaefer method.^42^ In short, a desiccant container filled with silica gel was weighed and placed on a holder a few millimeters on top of the subphase surface. The containers used were disposable desiccant cartridges (SP Industries, Warminster, PA), where the membrane was replaced with a Millipore Immobilon-P PVDF membrane with 450 nm pore size (Bedford, MA). After 10 min, the container was reweighed to determine how much water had evaporated from the subphase during the measurement. Each measurement was repeated a minimum of three times. A similar measurement was also done for the subphase without a lipid film, and using the obtained reference value, the evaporation resistances (*r*_m_) could be calculated according to eq 1.

1 $$r_{m}=A\left(W-W_{0}\right)dt\left(\frac{1}{M_{f}}-\frac{1}{M_{w}}\right)-\frac{\Delta m_{tot}t_{i}}{D{\rho t}_{tot}A_{trough}}$$

In the equation, *A* is the area of the desiccant container, *t* is measurement time and *W* and *W*_0_ are the equilibrium water vapor concentrations at the subphase and desiccant surfaces, respectively. The terms *M*_*f*_ and *M*_*w*_ are the masses of evaporated water through the lipid film and from a pure subphase surface, respectively. The last term ( $\frac{\Delta m_{tot}t_{i}}{D{\rho t}_{tot}A_{trough}}$ ) accounts for the drop in water level during the measurements. In the term, Δ*m*_tot_ is the total mass of evaporated water during the length of the experiment (*t*_tot_) and *t*_*i*_ is the time elapsed between measurement i and the initial control experiment. Finally, *D* is water’s diffusion constant in air, ρ is water’s density and *A*_trough_ is the surface area of the Langmuir trough. To evaluate the obtained results; differences between individual lipid species, as well as between C_15:0_/C_18:1_-OAHFA and octadecanol and their corresponding C_22:0_/C_18:1_-WE compositions, were assessed using two-sided Welch’s *t* tests,^43^ which were chosen to account for unequal variances.

## Results and Discussion

Promising chemical covers can be thought of as compositions that combine adequate spreading capabilities (in order to form a thin film across the surface of water bodies) and high evaporation resistance (in order to mitigate the loss of water due to evaporation).^16,17,44^ In previous work, we have explored the relationship between spreading capabilities and antievaporative features at conditions mimicking those of the ocular surface and found that it is indeed possible to tailor molecular solutions in order to maintain an appropriate balance between spreadability and antievaporative features in lipid films.^33,35,45^ Theoretical insights on the underlying dilemmas is available through previously developed concepts. In more detail, theories such as the energy barrier theory propose that monolayers inhibit evaporation by creating an energetic barrier that water molecules must overcome in order to “escape” from underneath.^42,46^ Other models, such as the density fluctuation theory^47,48^ and the accessible area theory^49^ suggest that evaporation may occur through potential openings or “holes” in the film structure. Overall, the current understanding indicates that densely packed liquid condensed and solid domains are required to efficiently retard water evaporation.^15,50^

These were thus the critical features that we sought to explore in this study. To accomplish this, we utilized a Langmuir trough system, which is ideal for mapping and comparing the baseline capabilities of distinct solutions under well controlled conditions. Water was selected as the subphase in this study because it is the most simplified aqueous phase available, ensuring repeatability between laboratories. For the temperature, room temperature was considered suitable because our previous work had focused on temperatures of 35 °C, and the temperature range of 25–35 °C can be considered as relevant from the perspective of water temperatures in open water systems. Additionally, room temperature is widely used for assessing the evaporation reduction of monolayers,^19,28,46,51–53^ and thus offers a comparable benchmark temperature.

In more detail, we focused on (1) recording surface pressure isotherms, in order to understand the phase transitions and packing behavior of the films; (2) imaging of the film structure by either BAM and/or FM in order to study the nature of the film structure; and, (3) assessing the evaporation rates of water through the distinct films applied in order to map their potential as chemical covers.

On a general level, when an amphiphilic surfactant is spread across a water surface, it lowers the surface tension, and this reduction is expressed as surface pressure. As the film is compressed, the available molecular area decreases, forcing the molecules closer together which leads to an increase in the surface pressure. As a consequence, the surface pressure–area isotherm describes surface pressure as a function of area/molecule, and provides valuable insight into the structure of a lipid film and potential phase transitions occurring as a function of surface pressure.^54^ Additional insights on film structure can be obtained through BAM/FM-imaging which help identify whether the film is in a liquid or solid phase and evaluate the cohesiveness of the film in general (including boundaries between different phases). The theoretical models conveyed above and findings from experimental work have suggested that antievaporative properties typically improve with increased surface pressure and a reduction in area/molecule, which leads to a more densely packed film, provided that the generated film remains stable. In the sections that follow, we will review our results systematically from this basis, starting with single components and finishing up with insights obtained from representative compositions.

### Assessing Film Properties of Individual Species

#### Surface Pressure Isotherms/Imaging of Film Structure

We started by analyzing the behavior of the individual lipid species. In the OAHFA series, both C_12:0_/C_18:1_-OAHFA and C_15:0_/C_18:1_-OAHFA spread effectively on the subphase surface and exhibited a clear phase transition between the liquid expanded and liquid condensed phases (Figure 2). Both species exhibited a relatively large surface pressure lift-off area (120 Å^2^/molecule for C_12:0_/C_18:1_-OAHFA and 110 Å^2^/molecule for C_15:0_/C_18:1_-OAHFA). This was interpreted in a similar fashion as previously, i.e., that at large areas the molecules lay flat on the subphase surface placing both the ester group and the carboxylic acid group in contact with the subphase (Figure 2, image I). During compression, the ester group detaches from the subphase, causing the hydrophobic parts of the molecules to orient away from water, while the carbon chains remain disordered (Figure 2, image II).^33,55^

**Figure 2:**
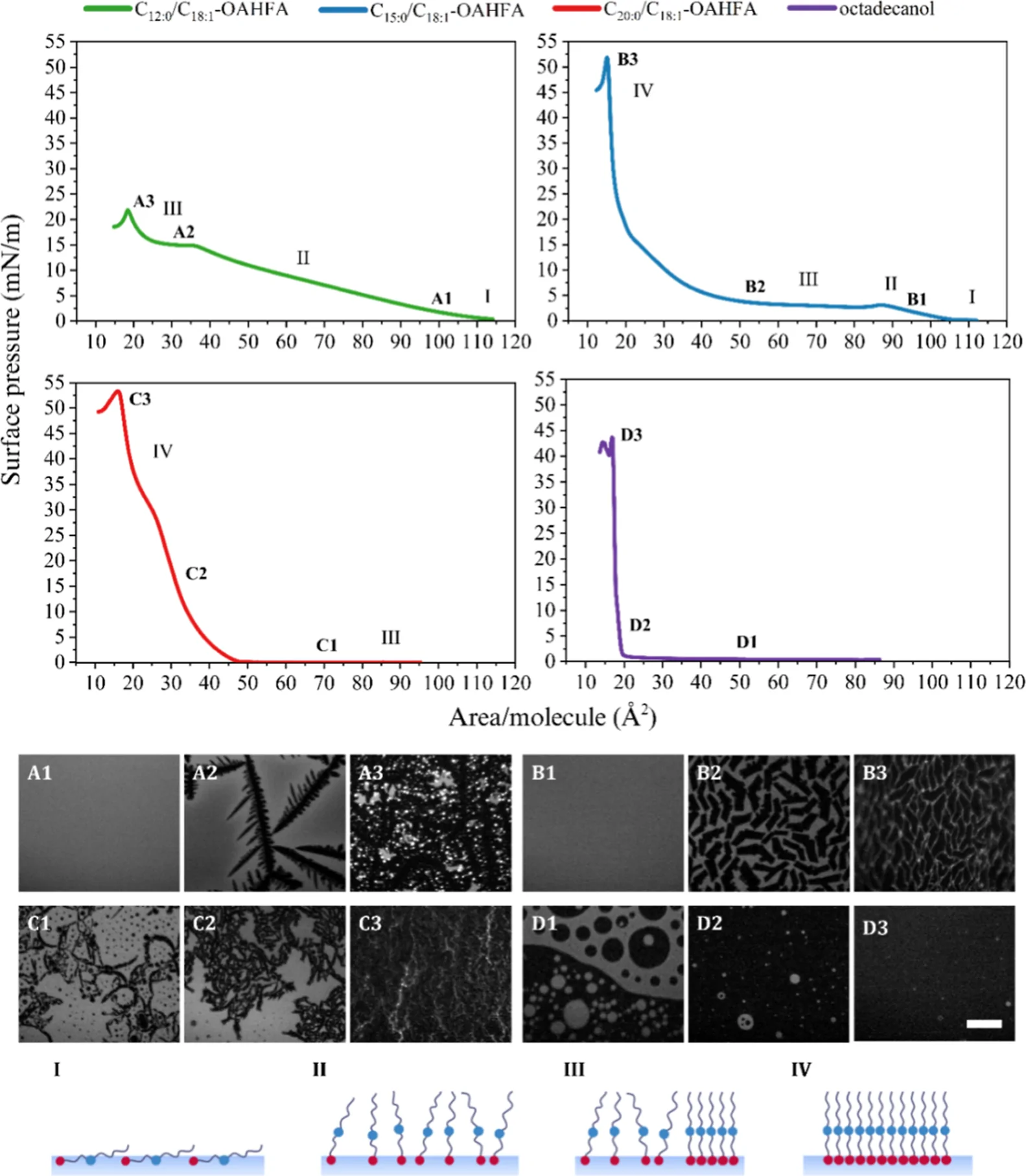
Surface pressure isotherms (as a function of area/molecule (Å^2^)) and FM-images of the studied OAHFAs and a component in a representative chemical cover in current use i.e. octadecanol. Images A1–A3 depict the film structures of C_12:0_/C_18:1_-OAHFA, images B1–B3 depict the film structures of C_15:0_/C_18:1_-OAHFA, images C1–C3 depict the film structures of C_20:0_/C_18:1_-OAHFA, and images D1-D3 depict the film structures of octadecanol. The scale bar corresponds to 100 μm. The images at the end, labeled I–IV, are a representative demonstration of how OAHFA molecules are likely to be arranged at different surface pressures. The red ball represents the carboxylic acid headgroup, and the blue ball represents the ester group.

Starting from areas of 35 Å^2^/molecule and 85 Å^2^/molecule a surface pressure plateau could be seen in the isotherms of C_12:0_/C_18:1_-OAHFA and C_15:0_/C_18:1_-OAHFA respectively, indicating a liquid expanded to liquid condensed phase transition, that resulted in a more dense and organized packing of the molecules (Figure 2, image III). As a result, the formation of dark crystalline areas (representing solid film structure) could be seen in the FM-images (Figure 2, images A2 and B2) and these regions originally coexisted with more fluid, disordered film regions (gray colored in the images). This is consistent with the behavior of NBD-labeled lipids, which exhibit higher fluorescence in fluid regions and reduced intensity in condensed, tightly packed areas.^56,57^ Upon a further increase in surface pressure, the solid domains in the film grew in size gradually. Based on the FM-images, the C_15:0_/C_18:1_-OAHFA film appeared fairly uniform at high surface pressures although minor fluid and disordered regions could be seen at the edges of the solid domains (Figure 2, image B3). On the other hand, C_12:0_/C_18:1_-OAHFA did not form a stable solid monolayer and the film collapsed at a relatively low surface pressure of ∼ 22–28 mN/m (Figure 2, Image A3). Thus, structural features such as the length of the parent chain, which affects properties such as the melting points, will heavily affect the behavior of the OAHFA films. This was known based on our previous studies and the reason why we screened several structural analogues herein as well.

In contrast to the two shorter OAHFAs discussed above, C_20:0_/C_18:1_-OAHFA displayed a condensed phase at the beginning of the measurement (Figure 2, image C1) and was not found to spread as efficiently as the other OAHFAs. During compression, the surface pressure remained close to zero until the area of 45 Å^2^, where the surface pressure began to rise steeply.

In the course of the surface pressure increase, the solid domains became more densely packed (Figure 2, image C2), and at an area of 25 Å^2^/molecule a kink was observed in the isotherm, most likely corresponding to a second order phase transition between two condensed phases. Even at high surface pressures, disordered interdomain regions remained visible in the film structure (Figure 2, image C3). This was interpreted as C_20:0_/C_18:1_-OAHFA forming aggregates (densely packed solid clusters) at the water surface instead of a homogeneous film.

In the case of octadecanol, the surface pressure isotherms and phase transitions have been studied extensively,^20,58–60^ and the previous findings could be confirmed also in the current studies. In short, octadecanol’s isotherm started at a transition into a liquid condensed phase (Figure 2, image D1), and according to the Harkins-Stenhagen nomenclature, this condensed film structure corresponds to the *L*_2_^′^ phase. The phase transition progressed with compression, and at the area of 18 Å^2^/molecule, a second phase transition was observed between the *L*_2_^′^ phase and the condensed LS phase, also known as the superliquid phase. These two condensed phases correspond to film structures with different organizations of the molecules. In the L_2_^′^ phase, the hydrophobic carbon chains tilt toward the next-nearest neighbor, while in the LS phase the chains are oriented normal to the surface.^59–61^

While both octadecanol and C_20:0_/C_18:1_-OAHFA existed initially in a condensed phase, the differences between the two are striking. For example, the FM-images revealed major heterogeneity in the film structure of C_20:0_/C_18:1_-OAHFA, whereas the film formed by octadecanol appeared to be more flexible and uniform at higher surface pressures, with only a few disordered regions, seen as bright speckles (Figure 2, image D3). This is likely due to the good spreading capabilities of octadecanol which has a high equilibrium spreading pressure of 35.2 mN/m at 25 °C.^18^ While we consider it unlikely that the fluorescent probe would have a major effect on the film structures, we cannot rule out the possibility of its cointegration into the film or other interactions which could contribute to the observed more fluid interdomain regions in the film structures. Therefore, we decided to investigate the evaporation resistance of the films in the absence of the fluorescent probe next, as this approach allows for a more definitive assessment of each lipid species potential for water evaporation mitigation.

#### Evaporation Resistance

It should be noted that the film structure and thus the evaporation resistance is a temperature dependent property and individual species should be tailored for distinct temperature ranges. In our previous reports, we have studied the innate properties of OAHFAs at physiological temperatures (35 °C) in order to understand their contributions to biological films.^33–35,45,62–64^ Herein, however, we studied the compounds at room temperature, as it offers a comparable reference temperature relevant to operating conditions in open water systems and provides a good indication on the requirement of molecular solutions within a relevant temperature range altogether. We observed that the correlation between film structure and evaporation resistance followed previously indicated trends, as showcased in Figure 3, and in all cases the maximum values were obtained when the surface pressure was high, the molecules densely packed and the film stable. Interestingly, the high surface pressures were reached at molecular areas smaller than those anticipated from the surface pressure isotherm measurements. This discrepancy was attributed to the intermittent pauses in compression during the resistance measurements. As expected, the shortest OAHFA, i.e. C_12:0_/C_18:1_-OAHFA, did not display a good evaporation resistance. The highest value obtained was only 0.2 s/cm (SD = 0.1) at an area of 3.5 Å^2^/molecule, which corresponds to an evaporation reduction of 9.8%. This is in line with the current understanding that an optimal monolayer requires both good spreading properties and a condensed structure.^16,44^ Although C_12:0_/C_18:1_-OAHFA demonstrated good spreadability, its evaporation resistance remained low, most likely due to its low film collapse surface pressure, which prevented the formation of a stable condensed structure.

**Figure 3:**
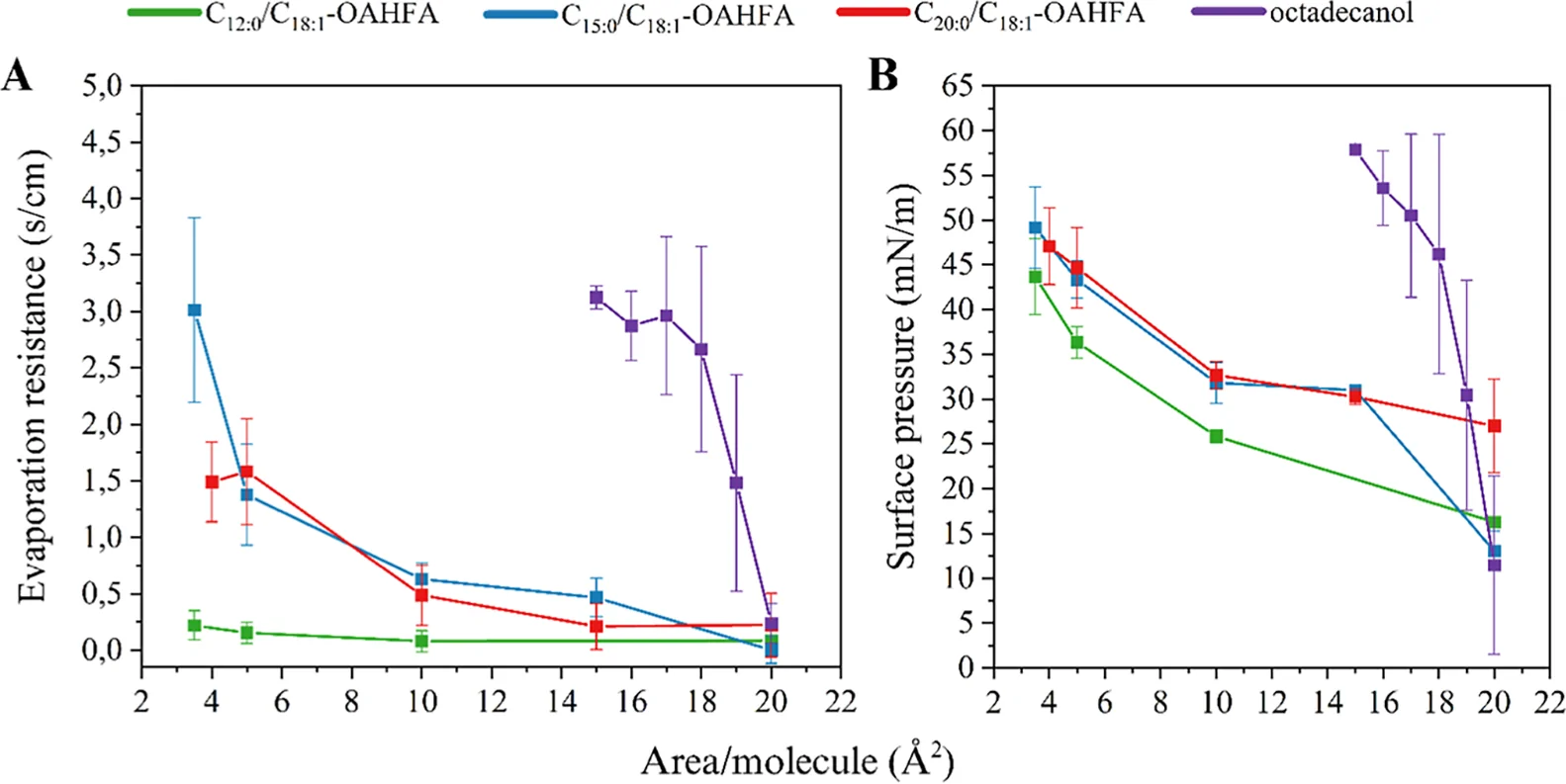
(A) Evaporation resistance (s/cm) as a function of area/molecule (Å^2^) for C_12:0_/C_18:1_-OAHFA, C_15:0_/C_18:1_-OAHFA, C_20:0_/C_18:1_-OAHFA and octadecanol at room temperature. (B) Surface pressure (mN/m) as a function of area/molecule (Å^2^) during the evaporation resistance measurements. The results present the average of a minimum of three measurements and the error bars depict the standard deviation.

Out of the studied OAHFAs, C_15:0_/C_18:1_-OAHFA reached the highest evaporation resistance, with the value increasing with compression and reaching an average of 3.0 s/cm (SD = 0.8) at an area of 3.5 Å^2^/molecule. This value was significantly higher than that of the C_12:0_/C_18:1_-OAHFA (*t* = 5.86, *p* = 0.025) and the C_20:0_/C_18:1_-OAHFA (*t* = 2.64, *p* = 0.073). The evaporation resistance of C_15:0_/C_18:1_-OAHFA corresponds to an evaporation reduction of 54% and is in the same range as the current golden standard component octadecanol which is utilized in products such as WaterSavr. In this case, the moderate evaporation resistance obtained for the compound derived from a combination of both adequate spreadability and the formation of a stable, fairly uniform and densely packed film.

In contrast to the predictions of the energy barrier theory,^46^ in which continuous extension of carbon chain lengths is connected to an ever increasing evaporation resistance, the evaporation resistance of C_20:0_/C_18:1_-OAHFA was inferior to that of C_15:0_/C_18:1_-OAHFA. In more detail, the highest value obtained for C_20:0_/C_18:1_-OAHFA was 1.6 s/cm (SD = 0.5) at an area of 5 Å^2^/molecule, which corresponds to a 35% decrease in water evaporation. Thus, chain lengths cannot be extended indefinitely with noted improvements in evaporation resistance. Moreover, and interestingly, the C_20:0_/C_18:1_-OAHFA was found to be superior to the shorter species in a previous study conducted at 35 °C.^33^ This indicates that the temperature is an important parameter to consider as it will affect the spreadability of the molecules, the coverage of the film and ultimately, the evaporation resistance reached. Overall, the results support the conclusion that an optimal evaporation resistant monolayer must be condensed and sufficiently fluid/flexible, implying that a compromise must be made between the theoretical maximum evaporation resistance and practical features such as spreadability.^16,44,65^

Lastly, the evaporation resistance of octadecanol was found to be 3.1 s/cm (SD = 0.1) at an area of 15 Å^2^/molecule which corresponds to an evaporation reduction of 57% and is in line with previously published values.^19,20^ There was no statistically significant difference between the evaporation resistance values of octadecanol and those obtained for C_15:0_/C_18:1_-OAHFA (*t* = 0.24, *p* = 0.83) whereas C_12:0_/C_18:1_-OAHFA and C_20:0_/C_18:1_-OAHFA were clearly inferior (*t* = −31.73, *p* = < 0.0001 and *t* = −5.63, *p* = 0.026 respectively). Based on this assessment, we concluded that OAHFAs may challenge the current gold standard in the field. Yet, the external temperature will need to be carefully considered as this will influence the properties displayed by the lipid films as such and distinct solutions will most probably need to be tailored based on the external temperature receding at the water source to protected from evaporation. Moreover, other factors, such as costs, ease of applicability, contact time, administration frequency and environmental fate (degradation products, effects on water pH etc.) will also need to be considered in more depth as part of future work. Some examples are warranted: while fatty acids have been reported to exhibit high evaporation resistances, achieving effective spreading without the need for solvent assistance may pose a challenge to be overcome when aiming at their use in water reservoirs.^17^ On the other hand, the carboxylic acid headgroup is more hydrophilic than the hydroxyl group in fatty alcohols, which may enhance the contact time of these molecules at the water surface thereby potentially extending the monolayer lifespan.^51^ Both these factors, and several others, will be interesting to explore in more detail going forward. Within the confinements of the current laboratory-scale assessment and pursuit of new molecular solutions for water evaporation mitigation, we next focused on the assessment of representative lipid compositions as our previous work had showed that tailored compositions may display important improvements over single components as such.

### Assessing Film Properties of Representative Lipid Compositions

#### Surface Pressure Isotherms/Imaging of Film Structure

In our previous work, we have shown that the combination of OAHFAs and WE can lead to films with superior evaporation resistant properties over individual species alone.^34^ It has been noted in other cases as well that mixing lipid species in specific compositions can lead to an increase in the evaporation resistance,^66,67^ but lipid compositions can also display reduced evaporation resistant properties.^50^ Therefore, we herein proceeded to study representative compositions featuring the most promising individual compounds identified (at room temperature), i.e. C_15:0_/C_18:1_-OAHFA and octadecanol together with a chosen model WE (C_22:0_/C_18:1_-WE). While we employed similar techniques as described above, the compositions were also imaged with BAM to gain additional information on the film structures. In Langmuir experiments, WEs (including C_22:0_/C_18:1_-WE), tend to spread poorly and form aggregates when the interface temperature is below their melting point,^68,69^ requiring to be accompanied by polar lipids in order to improve spreadability and film stability.^69^ Similar observation could be made in this work as well. When assessing the behavior of C_22:0_/C_18:1_-WE on its own, it was found that the compound did not spread efficiently, but instead formed disordered aggregates, seen as bright regions in FM-images (data not shown).

We proceeded by studying a 1:1 C_15:0_/C_18:1_-OAHFA:C_22:0_/C_18:1_-WE composition. The C_22:0_/C_18:1_-WE was found to aggregate at the start of the measurements also in the composition, which could be seen both in the FM and BAM images, when comparing them to those obtained for the C_15:0_/C_18:1_-OAHFA itself (Figure 4, image A1). In addition, the initial lift-off area of the composition was approximately half that of pure C_15:0_/C_18:1_-OAHFA. This suggests that at low surface pressures, C_15:0_/C_18:1_-OAHFA resided at the air–water interface while the majority of C_22:0_/C_18:1_-WE was excluded from the layer in direct contact with the aqueous phase. Upon compression, the surface pressure isotherm exhibited a clear phase transition starting from an area of ∼ 50 Å^2^/molecule and the FM-images showcased the formation of dark condensed domains (Figure 4, image A2). As the film was further compressed, the size of these condensed regions grew, and at high surface pressures the film appeared to be a combination of both dark-colored ordered regions and light-colored more fluid regions (Figure 4, images A3 and A4). In the BAM-images, however, the film appeared to be more uniform with some bright spots most likely indicating the presence of C_22:0_/C_18:1_-WE aggregates on top of a densely packed solid film.

**Figure 4:**
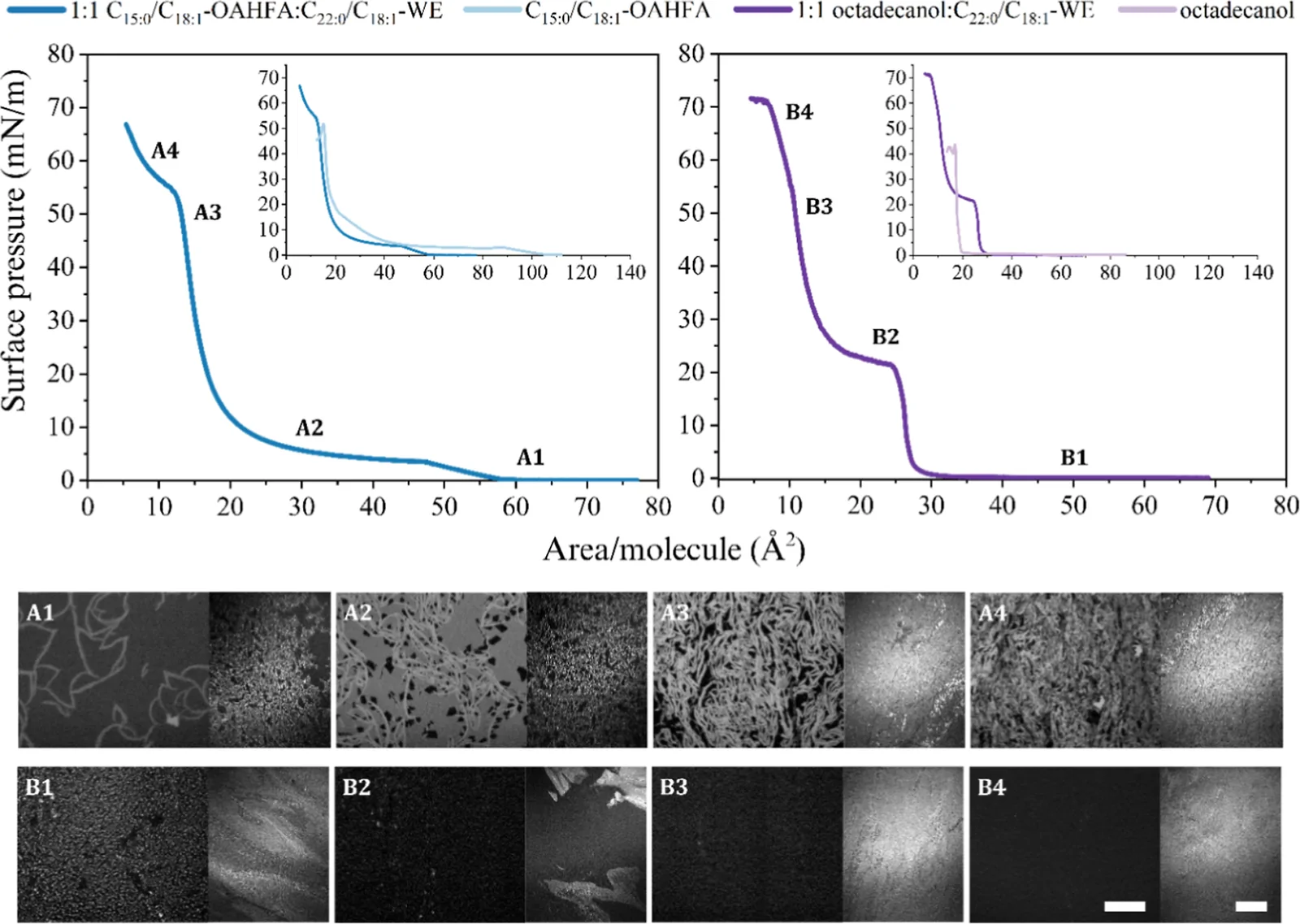
Surface pressure isotherms (as a function of area/molecule (Å^2^)) and FM-images (on the left) and BAM-images (on the right) of the representative lipid compositions. The surface pressure isotherms for the pure compounds are shown for comparison. Images A1–A4 depict the film structures of 1:1 C_15:0_/C:_18:1_-OAHFA:C_22:0_/C_18:1_-WE and images B1–B4 depict the film structures of 1:1 octadecanol/C_22:0_/C_18:1_-WE. The scale bar in the FM- and BAM-images depicts 100 μm and 1 mm, respectively.

Simultaneously with the progression of the phase transition and increase in surface pressure, the mean molecular area approached that of pure C_15:0_/C_18:1_-OAHFA, remaining only slightly smaller at high surface pressures. This convergence in molecular area was interpreted so that the majority of the C_22:0_/C_18:1_-WE molecules gradually incorporated into the interfacial lipid layer and that this incorporation was tied to the phase transition. Thus, in other words, the C_15:0_/C_18:1_-OAHFA was capable of spreading the C_22:0_/C_18:1_-WE to a certain extent. That being said, the persistence of phase-separated structures in both the FM and BAM images along with the smaller molecular area than that of pure C_15:0_/C_18:1_-OAHFA, indicates that C_22:0_/C_18:1_-WE did not fully integrate into the monolayer. Notably, the incorporation took place to a lesser extent than previously noted upon with a longer OAHFA species.^34^ This distinct behavior may suggest that longer OAHFAs, such as the naturally occurring ones, may be better suited for incorporation of other lipids into the polar part of the lipid film, which is in contact with the aqueous subphase. However, the effects of temperature cannot be ruled out based on the screening conducted herein and thus more work will be required later on to address these aspects in more detail.

In the case of the 1:1 octadecanol/C_22:0_/C_18:1_-WE composition, areas of different intensities and both dark condensed regions and disordered bright speckles were seen in the film at low surface pressures (Figure 4, image B1). Interestingly, the lift-off area of the composition (30 Å^2^/molecule) occurred at a larger value than in octadecanol (20 Å^2^/molecule). This observation was interpreted in such a way that octadecanol spread C_22:0_/C_18:1_-WE in a bulky V-shaped conformation, which would be in line with the previous suggestions on the conformations adopted by this species and other WEs in expanded monolayers.^68,70^

At the lift-off area, the size of the disordered regions diminished substantially (Figure 4, image B2), and at high surface pressures the film appeared to be condensed and highly uniform in the FM-images (Figure 4, images B3 and B4). While discussing the film structure of the composition, we would like to note that in the surface pressure range of 20–25 mN/m, a plateau can be seen in the isotherm, and the BAM-images are suggestive of the formation of a multilayered film (Figure 4, image B2). Since no other structural changes are apparent in the images, it seems likely that these changes correspond to a partial collapse of the less polar C_22:0_/C_18:1_-WE. This would suggest that at high surface pressures the film consisted of two components: (1) an interfacial layer in contact with the subphase, largely consisting of octadecanol and (2) a layer of collapsed WE residing on top of this underlying layer. Additional studies with synchrotron sources would be required to fully understand the interactions taking place between the species in compositions and the full effects on the packing, tilt angles and location of distinct species in the overall film structure but these investigations were considered to be beyond the scope of this work. We herein continued with investigating evaporation resistant properties of these lipid compositions.

#### Evaporation Resistance

Previously, the integration of C_22:0_/C_18:1_-WE into the film formed by OAHFAs resulted in a considerable increase in evaporation resistance.^34^ In this study, the characterization of film properties suggested that at high surface pressures C_22:0_/C_18:1_-WE could integrate into C_15:0_/C_18:1_-OAHFA, whereas octadecanol supported the integration of the WE to a lesser extent. Nevertheless, and to our surprise, the maximum evaporation resistance was significantly improved in a 1:1 octadecanol/C_22:0_/C_18:1_-WE composition when compared to the values obtained for octadecanol (*t* = 12.6, *p* = 0.006). A similar trend could also be identified in the 1:1 C_15:0_/C_18:1_-OAHFA:C_22:0_/C_18:1_-WE composition when compared to C_15:0_/C_18:1_-OAHFA alone, although the beneficial effects and maximum evaporation resistance reached were not as high as for the fatty alcohol and wax ester composition. In more detail, the maximum evaporation resistance for the 1:1 C_15:0_/C_18:1_-OAHFA:C_22:0_/C_18:1_-WE composition was found to be 4.7 s/cm (SD = 0.6), which corresponds to a 66% reduction in water evaporation, whereas it was 12 s/cm (SD = 1.2) for the 1:1 octadecanol/C_22:0_/C_18:1_-WE composition, which corresponds to a 84% reduction in water evaporation (Figure 5).

**Figure 5:**
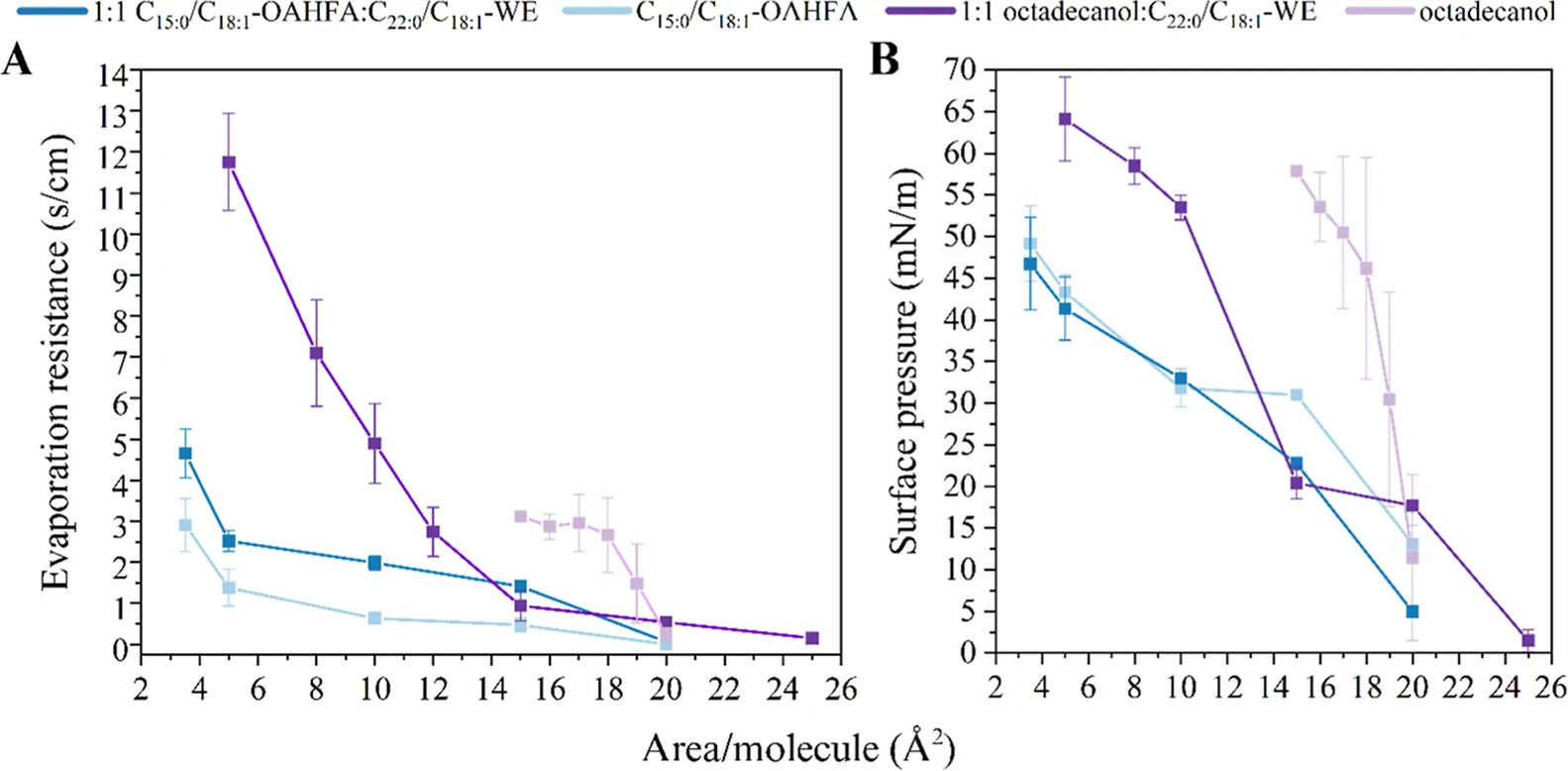
(A) Evaporation resistance (s/cm) as a function of area/molecule (Å^2^) for 1:1 C_15:0_/C_18:1_-OAHFA:C_22:0_/C_18:1_-WE and octadecanol/C_22:0_/C_18:1_-WE compositions. (B) Surface pressure (mN/m) as a function of area/molecule (Å^2^) during the evaporation resistance measurements. The results present the average of a minimum of three measurements, and the error bars depict the standard deviation. The values for the pure substrates are shown for comparison.

Thus, the beneficial effect of including the C_22:0_/C_18:1_-WE seems clear in terms of improved evaporation resistance. It has been noted, that binary surfactant systems can show superior performance to single component monolayers in specific compositions,^34,66,67^ with enhanced evaporation resistance attributed to improved packing and enhanced stability and spreading.^67^ Consistent with this, we observed that the addition of C_22:0_/C_18:1_-WE increased the collapse surface pressures of the compositions (Figures 2 and 4), suggesting a stabilizing effect on the films. While surface pressure isotherms provide some insight into the spreading behavior of the individual species and compositions, a more detailed characterization of spreading properties, such as equilibrium spreading pressures, was not performed here and would require dedicated experiments beyond the scope of this work.

Returning to the significance of the evaporation resistance values uncovered: while the maximum values are obtained at higher surface pressures than those typically observed in water reservoirs, they nevertheless show that there are multiple possibilities for significantly improving the baseline properties of compounds and compositions utilized as chemical covers in water evaporation mitigation. The maximum values uncovered here are comparable to the best physical covers employed,^5^ which suggests that these types of solutions may become highly potent and appealing for water evaporation mitigation in the future. While the Langmuir system provides valuable baseline insights and is essential to the assessment and development of improved chemical covers, it does not model environmentally relevant conditions which would be expected to affect the performance of the lipid film in a real-world scenario. Some factors were addressed above at the end of the section on individual species. Others include environmental parameters such a wind, humidity, and even the composition of water in reservoirs (including organic material which may disturb film formation) and thus future outdoor field studies in open water tanks or evaporation pans will be required to aid in translation of these promising early stage laboratory-scale findings toward the intended applications.

## Conclusions

The global climate change is expected to increase the devastating effects of drought on a worldwide scale, and some of the key challenges which societies may face are related to water conservation. Chemical covers represent one possibility for reducing water evaporation in water systems and open water bodies. In this work, we set out to perform a laboratory-scale assessment of the possibilities of tailoring improved chemical covers based on OAHFAs, fatty alcohols or compositions featuring WEs. The laboratory-scale assessment exceeded our expectations, and several promising solutions could be identified with an improved evaporation resistance in comparison to current state-of-the-art chemical covers. Moreover, the studies were here performed at room temperature which complemented our previous work in the field at which we had explored the antievaporative features of tear film lipids and related compositions at ocular surface conditions and temperatures of 35 °C. Combining the insights from this work and our previous studies on structural analogues of tear film lipids/compositions, it becomes clear that components utilized in antievaporative lipid films need to be tailored for specific temperatures. At higher temperatures, species with higher melting point (e.g., species with longer chain lengths) are more viable options whereas at lower temperatures, such as room temperature, species with lower melting points (e.g., species with shorter chain lengths) should serve as a base. Overall, both our previous work and the current manuscript identifies compositions which will function optimally in the temperature range of 25–35 °C which we consider to be a relevant interval from the water conservation perspective. Our promising findings will next need to be verified in outdoor studies utilizing evaporation pans or open water tanks in order to assess the potential of these chemical covers in a setting that more closely resembles a real-world scenario and to explore the effect of environmental conditions (temperature, wind etc.), head groups, and possible degradation pathways on their contact time and performance. Simultaneously, the compounds and their degradation products will need to be carefully reviewed from an ecological perspective. Encouraged by our findings, we look forward to continued explorations on this matter and will report on our findings from the following stages in due course.
